# Atrial fibrillation–induced neurocognitive and vascular dysfunction is averted by mitochondrial oxidative stress reduction

**DOI:** 10.1172/jci.insight.189850

**Published:** 2025-10-07

**Authors:** Pavithran Guttipatti, Ruiping Ji, Najla Saadallah, Uma Mahesh R. Avula, Deniz Z. Sonmez, Albert Fang, Eric Li, Amar D. Desai, Samantha Parsons, Parmanand Dasrat, Christine Sison, Yanping Sun, Chris N. Goulbourne, Steven R. Reiken, Elaine Y. Wan

**Affiliations:** 1 Division of Cardiology, Department of Medicine, Vagelos College of Physicians and Surgeons, Columbia University, New York, New York, USA.; 2 Department of Medicine, University of Mississippi, Jackson, Mississippi, USA.; 3 Oncology Precision Therapeutics and Imaging Core, Herbert Irving Comprehensive Cancer Center, Columbia University, New York, New York, USA.; 4 Center for Dementia Research, Nathan S. Kline Institute for Psychiatric Research, Orangeburg, New York, USA.; 5 Department of Physiology and Cellular Biophysics and Clyde & Helen Wu Center for Molecular Cardiology, Vagelos College of Physicians and Surgeons, Columbia University, New York, New York, USA.

**Keywords:** Cardiology, Vascular biology, Arrhythmias, Ion channels

## Abstract

Atrial fibrillation (AF) is a prevalent arrhythmia with known detriments such as heart failure, stroke, and cognitive decline even in patients without prior stroke. The mechanisms by which AF leads to cognitive dysfunction are yet unknown, and there is a lack of animal models to study this disease process. We previously developed a murine model of spontaneous and prolonged episodes of AF, a double transgenic mouse model with cardiac-specific expression of a gain-of-function mutant voltage-gated sodium channel (DTG-AF mice). Herein, we show, for the first time to our knowledge, a murine model of AF without any cerebral infarcts exhibiting cognitive dysfunction, including impaired visual learning and cognitive flexibility on touch screen testing. Mesenteric resistance arterial function of DTG-AF mice showed significant loss of myogenic tone, increased wall thickness and distensibility, and mitochondrial dysfunction. Brain pial arteries also showed increased wall thickness and mitochondrial enlargement. Furthermore, DTG-AF mice have decreased brain perfusion on laser speckle contrast imaging compared with controls. Cumulatively, these findings demonstrate that AF leads to vascular structural and functional alterations necessary for dynamic cerebral autoregulation, resulting in increased cerebral stress and cognitive dysfunction. Expression of mitochondrial catalase (mCAT) to reduce mitochondrial reactive oxygen species (ROS) was sufficient to prevent vascular dysfunction due to AF, restore perfusion, and improve cognitive flexibility.

## Abstract 2

A murine model of atrial fibrillation exhibits cognitive and vascular dysfunction which is prevented by expression of mitochondrial catalase to reduce mitochondrial reactive oxygen species.

## Graphical abstract

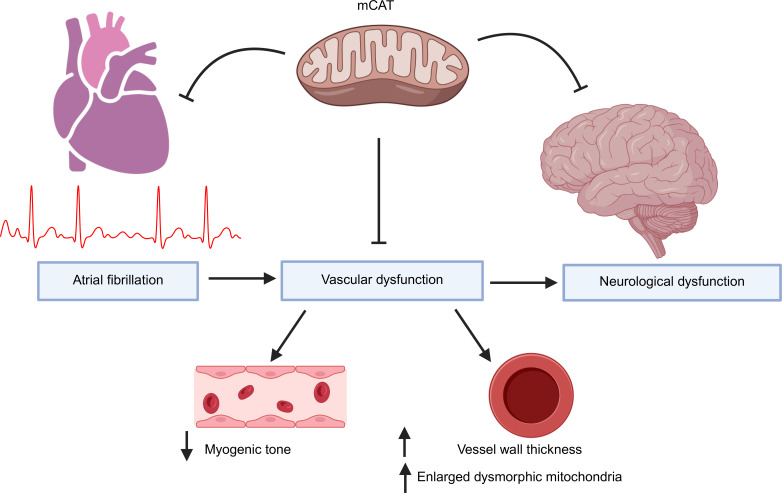

## Introduction

AF is the most common arrhythmia seen in clinical practice, and its treatment is important for prevention of heart failure as well as thromboembolic complications such as stroke. There is increasing evidence that the presence of AF increases the risk of developing cognitive impairment, cognitive decline, and dementia independent of stroke (1–3). AF has been associated with worsened learning and memory performance, deficits in attention and executive functions (4), and increased risk of dementia (5–8). Previous studies have demonstrated an association between AF and neurological decline even after correcting for such confounders such as age, hypertension, and diabetes (5–7). The molecular mechanisms by which AF leads to cognitive dysfunction are unclear, and there is lack of an animal model to recapitulate the AF-induced cognitive dysfunction seen in humans. Clinical studies in humans are also hindered due to the prolonged duration of time needed to follow up with patients to assess the development of cognitive dysfunction due to AF.

We have previously established a mouse model of persistent Na^+^ current that demonstrates spontaneous and prolonged episodes of AF, with structural and electrophysiological dysfunction similar to patients with AF (9, 10). This murine model exhibits a cardiac-specific sodium channel mutation with sustained episodes of AF at an early age. Implanted s.c. electrocardiogram (ECG) telemeters showed spontaneous AF in nonanesthetized mice with an average AF burden of 35%–37% ± 10% during a 20-hour period, which was never observed in nontransgenic or single transgenic littermates implanted with ECG telemeters. The longest continuous episode of AF observed was 1 hour, 52 minutes (9, 11). We utilized this double transgenic murine model of AF (DTG-AF), to study the effect of AF on cognition, brain structure, and vascular function. Our prior work has demonstrated the role of reactive oxygen species (ROS) in the pathogenesis of AF, and attenuating mitochondrial oxidative stress by expressing the human catalase protein in mitochondria (mitochondrial catalase [mCAT]) led to a 95% reduction in AF burden in mice (11). Here we investigate if reduction in mitochondrial oxidative stress and AF burden via mCAT is sufficient to reverse cognitive decline, offering potentially new therapeutic targets for AF-induced cognitive dysfunction.

## Results

### Impaired visual discrimination learning and cognitive flexibility in DTG-AF mice.

**Figure 1:**
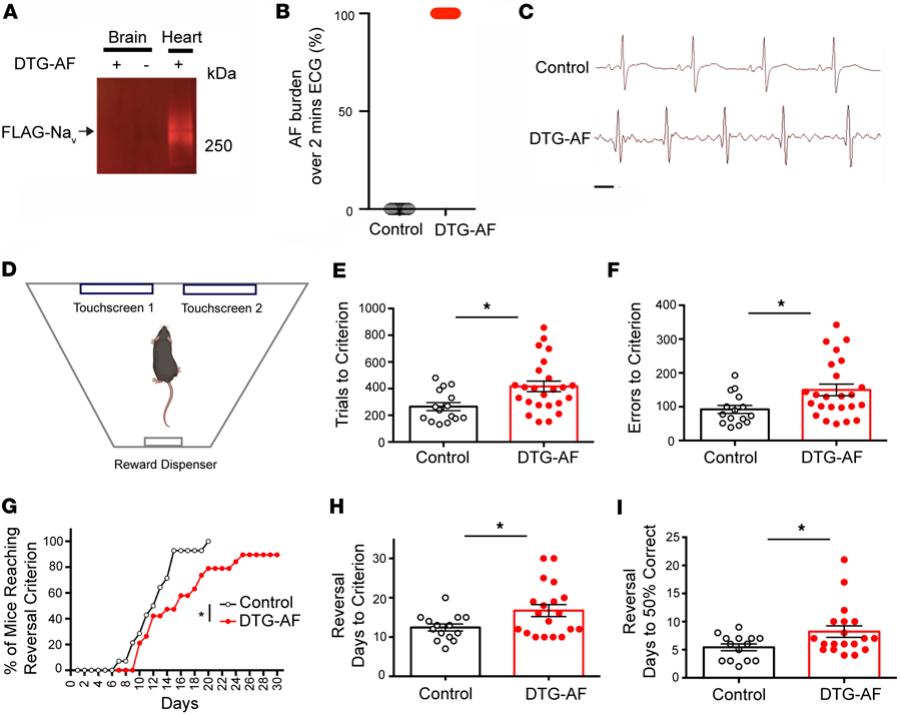
Impaired visual learning and cognitive flexibility in DTG-AF mice. (**A**) Transgenic NaV1.5 channels are not expressed in brain. Representative anti-FLAG antibody immunoblot of homogenates from brain and heart of DTG-AF compared with control (–). (**B**) Limb lead ECG 2 minute recording shows control mice (*n* = 12) has 0% AF burden, whereas DTG-AF (*n* = 15) mice have 100% AF burden. (**C**) Representative limb lead ECG tracings of control and DTG-AF mice. Scale bar: 100 ms. (**D**) Schematic depiction of mouse in touch screen testing apparatus from above… (**E**) DTG-AF mice require significantly greater trials to achieve criterion performance than control (unpaired 2-tailed *t* test, *P* < 0.05) (*n* = 15 control, *n* = 24 DTG-AF mice). (**F**) DTG-AF make significantly greater errors when learning. (**G**) Percentage of mice reaching criterion each day after the correct stimulus is reversed. DTG-AF perform significantly worse than control mice (log-rank Mantel-Cox, χ^2^ = 5.42, *P* = 0.02) (*n* = 14 control, *n* = 19 DTG-AF). (**H**) DTG-AF mice require more days to learn the reversed stimulus than control (*P* < 0.05). (**I**) Analysis of early reversal learning shows DTG-AF mice require more days to reach 50% chance performance than control (*P* < 0.05). Data are shown as mean ± SEM, *P < 0.05.

Our DTG-AF mice express a FLAG-tagged mutated human NaV1.5 channel with an Ala-substitution at position F1759, under a modified heart-specific α-MHC promoter that leads to expression in the heart but not brain as confirmed by anti-FLAG antibody in 5 mice (Figure 1A). Cardiac expression of mutated NaV1.5 in DTG-AF mice leads to substantial AF (Figure 1, B and C). To assess cognitive function in DTG-AF mice, we used the automated touch screen test to assess visual discrimination and learning (12). The touch screen test offers a method to assay perceptual processing and learning of stimulus-reward relationships, and it has potential for translation, given similarity to human cognitive tests such as the Cambridge Neuropsychological Test Automated Battery (CANTAB) (13). After an initial pretraining phase, mice are placed in a chamber where they are presented with 2 different visual stimuli on touch screens (Figure 1D). Upon selection of the correct stimuli a reward is presented, while incorrect responses lead to a correction trial where stimuli are represented until a correct response is achieved. Two- to 8-month-old male and female mice were subjected to 30 trials daily until they reach a success criterion of 80% initial correct responses over 30 trials for 2 consecutive days. DTG-AF mice required a significant increased number of trials by 57% to reach criterion compared with littermate single transgenic and transgene negative control mice, henceforth referred to as controls (Figure 1E) (mean 265 ± 30 versus 417 ± 40), suggesting deficits in acquisition of learning. DTG-AF mice also made 63% more errors before reaching criterion (Figure 1F) (92 ± 12 versus 150 ± 17), took 41% more days to reach criterion (Supplemental Figure 1, A and B; supplemental material available online with this article; https://doi.org/10.1172/jci.insight.189850DS1) (11.6 ± 1.7 versus 16.4 ± 1.7), and needed 50% more correction trials (Supplemental Figure 1C) (190 ± 28 versus 285 ± 32). The touch screen test also assessed other metrics of cognition, such as number of inactive touches made during screen time-out periods between trials indicative of perseverative behavior. A perseveration index was also calculated based on the number of correction trials a mouse experiences per error. However, DTG-AF mice did not show changes in inactive touches or perseveration index compared with controls (Supplemental Figure 1, D and E). The latency regarding collection of a reward after a correct response was also not significantly different between groups (Supplemental Figure 1F), although the spread in the controls may prevent interpretation.

After initial learning, the touch screen test was used to assess cognitive flexibility by reversing the correct stimulus that provides reward (12–14). Analysis of percentage of mice reaching the criterion of 80% accuracy with the reversed stimulus per day demonstrated DTG-AF mice performing significantly worse than controls (Figure 1G) and requiring more days to learn the new stimulus (Figure 1H) (12.4 ± 0.9 versus 16.7 ± 1.5). The number of trials, errors, and correction trials was not significantly different between DTG-AF and control mice in reversal (Supplemental Figure 2). We also conducted a further analysis of the days required for mice to achieve 50% accuracy, representative of the early stage of retraining when the original stimulus is unlearned and chance level reached (15, 16). Congruently, DTG-AF mice required 1.5 times the days to unlearn the original stimulus (Figure 1I) (5.4 ± 0.6 versus 8.2 ± 1.0). Taken together, these data suggest that DTG-AF mice show cognitive impairments in the domain of visual learning, along with reduced cognitive flexibility and learning adaptability.

### Ventriculomegaly and increased left frontal lobe diffusion in DTG-AF mouse brains.

**Figure 2:**
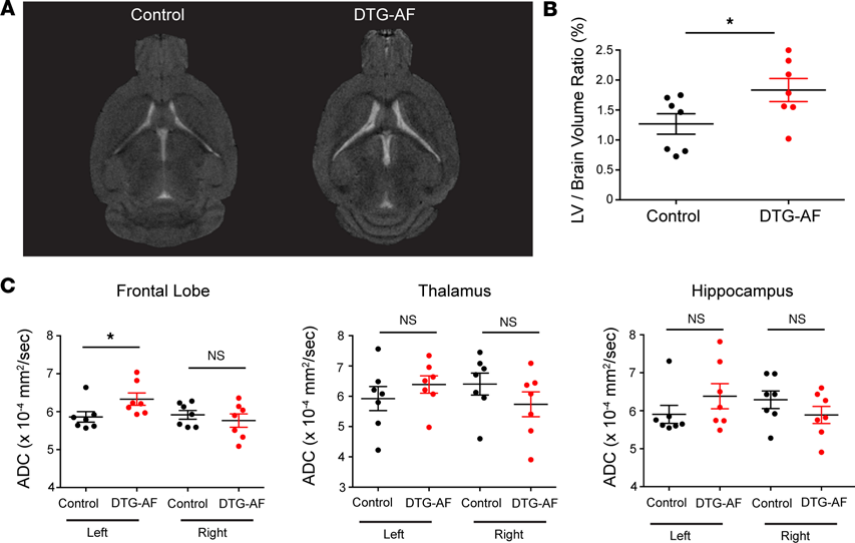
Ventriculomegaly and increased ADC in left frontal lobe of DTG-AF mice on brain MRI. (**A**) Representative T2-weighted axial brain MRI images of control and DTG-AF mice demonstrating lateral ventricle enlargement. (**B**) Quantification of lateral ventricle (LV) brain volume ratio shows ventriculomegaly in DTG-AF mice. Unpaired 2-tailed *t* test, *P* < 0.05 (*n* = 7 control, *n* = 7 DTG-AF mice). (**C**) Average ADC (apparent diffusion coefficient) values in the frontal lobe, thalamus, and hippocampus for the left and right hemisphere from diffusion weighted imaging. Left frontal lobe of DTG-AF mice shows greater diffusion than in control mice. Unpaired 2-tailed *t* test, *P* < 0.05 (*n* = 7 control, *n* = 7 DTG-AF mice). Data are shown as mean ± SEM, *P < 0.05.

Given the cognitive deficits observed in DTG-AF mice, we investigated whether cerebral imaging would reveal infarcts similar to the increased asymptomatic silent cerebral infarcts (SCIs) seen in patients with AF (17, 18). T2-weighted MRI and diffusion weighted imaging (DWI) of controls, and DTG-AF mice did not reveal any hypointense or hyperintense lesions that could be consistent with infarcts. However, DTG-AF mice did have significantly enlarged lateral ventricle volumes normalized to brain size (Figure 2, A and B) (1.27 ± 0.17 versus 1.83 ± 0.19). This has been similarly reported in older patients with AF, whom have been shown to have significantly faster annual increase in lateral ventricle volume than patients without AF, independent of cerebral infarcts (19). We additionally quantified apparent diffusion coefficient (ADC) in different brain regions from DWI. Clinically, reduced ADC is seen in the acute setting at sites of ischemic stroke due to loss of Na^+^/K^+^-ATPase activity and cell swelling that restricts water diffusion (20). However, ADC has also been studied chronically in aging, with some analyses reporting increased ADC in older patients while other studies report constant values (21–24). We found that the DTG-AF mice showed significantly increased ADC in the left frontal lobe compared with controls (5.86 × 10^–4^ ± 0.14 × 10^–4^ versus 6.33 × 10^–4^ ± 0.16 × 10^–4^ mm^2^/sec), while other brain regions such as the thalamus and hippocampus did not (Figure 2C). While the mechanism behind increased ADC is unclear, it has been suggested that structural changes that allow for increased water movement, such as loss of myelination or blood-brain barrier damage leading to vasogenic edema, may be involved (21, 25). We additionally performed brain perfusion imaging using dynamic T2-weighted contrast-enhanced MRI but did not note any significant differences between controls and DTG-AF mice (not shown). Given the lack of infarcts on brain imaging, we investigated other mechanisms that may explain cognitive decline in our animal model.

### Vascular structural and ultrastructural remodeling in DTG-AF mice.

**Figure 3:**
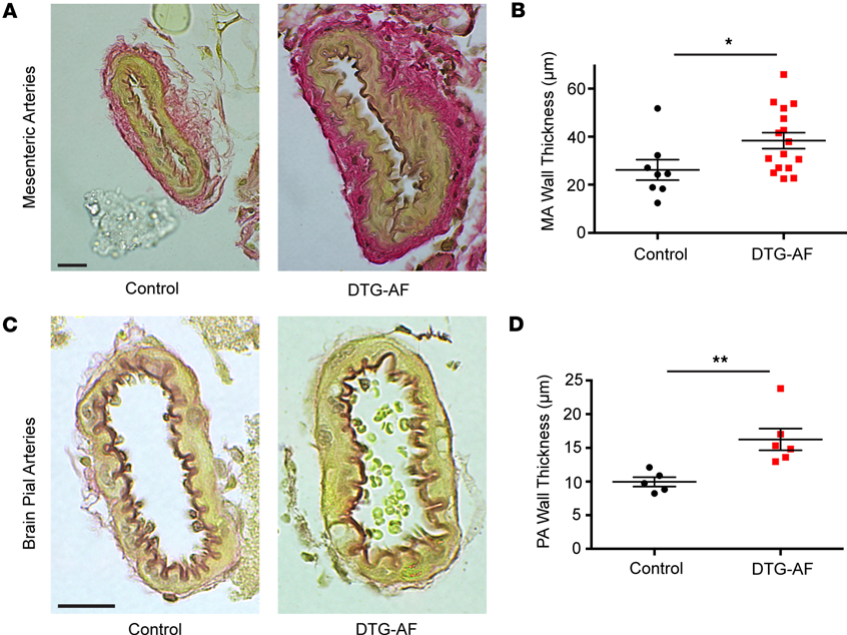
Vascular remodeling in DTG-AF brain and pial arteries. (**A** and **B**) Elastin-stained transverse sections of mesenteric arteries (MA) demonstrating increased wall thickness and enlarged tunica media in DTG-AF mice compared with controls (unpaired 2-tailed *t* test, *P* < 0.05) (*n* = 8 control vessels from 5 mice, *n* = 16 DTG-AF vessels from 6 mice). Scale bar: 20 μm. (**C** and **D**) Brain pial arteries (PA) show increased wall thickness in DTG-AF mice than control on histology (*P* = 0.01) (*n* = 5 control vessels from 3 mice, *n* = 6 DTG-AF vessels from 2 mice). Scale bar: 20 μm. Data are shown as mean ± SEM, *P < 0.05, **P < 0.01.

**Figure 4:**
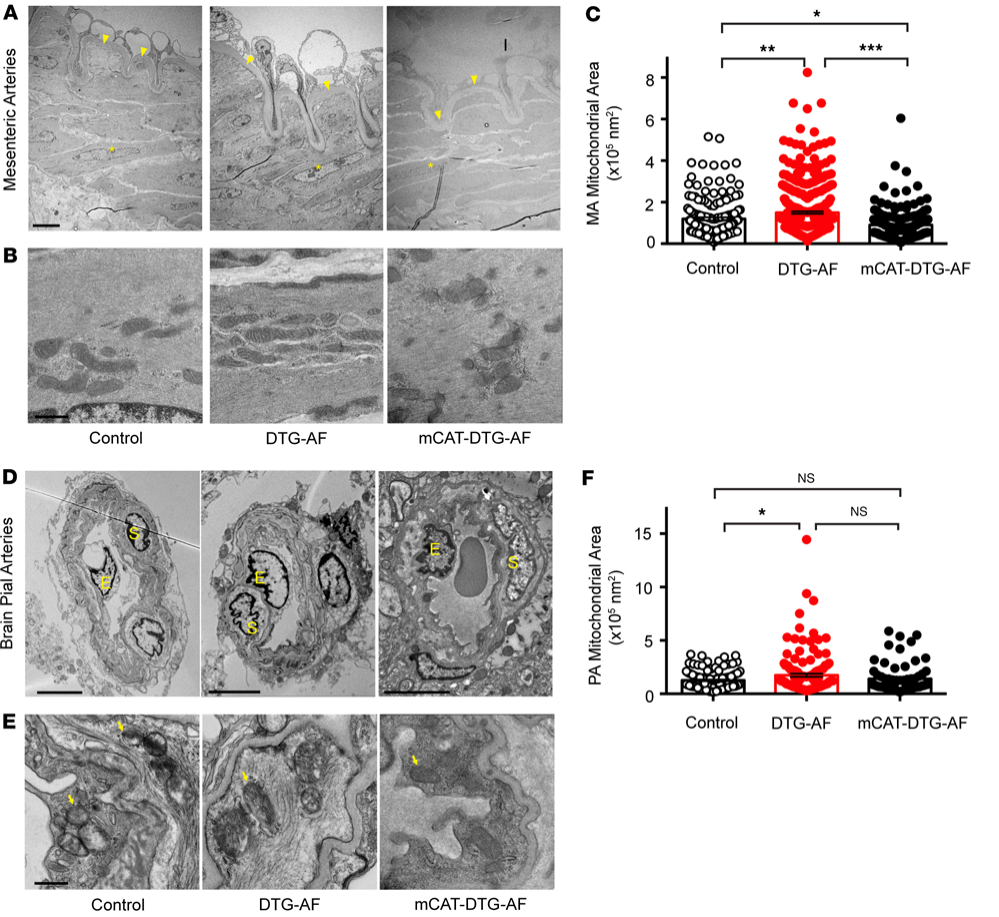
Vascular ultrastructural alterations in DTG-AF vessels. (**A**) Transmission electron microscopy (TEM) of mesenteric arteries in cross section with internal elastic lamina (yellow arrowhead) and smooth muscle cell layers (asterisk) labeled. Scale bar: 5 μm. (**B**) Higher magnification of mesenteric arteries with mitochondria visualized surrounded by actin filaments. DTG-AF vessels show enlarged mitochondria, while expression of mCAT results in smaller mitochondria. Scale bar: 500 nm. (**C**) Quantification of individual mitochondrial size in mesenteric arteries (1-way ANOVA with Tukey’s multiple-comparison test, *n* = 168 control mitochondria from *n* = 3 mice, *n* = 440 DTG-AF mitochondria from *n* = 5 mice, *n* = 171 mCAT-DTG-AF mitochondria from *n* = 2 mice). (**D**) TEM of cross-sections of brain pial arteries showing normal architecture with endothelial cell nucleus (labeled as “E”) and smooth muscle cell nucleus (labeled as “S”) shown. Scale bar: 5 μm. (**E**) Higher magnification of brain pial arteries demonstrating enlarged mitochondria in DTG-AF vessels compared with control. Arrows demarcate enlarged mitochondria. Scale bar: 500 nm. (**F**) Quantification of individual mitochondrial size in pial arteries (1-way ANOVA with Tukey’s multiple-comparison test, *n* = 142 control mitochondria from *n* = 3 mice, *n* = 160 DTG-AF mitochondria from *n* = 5 mice, *n* = 69 mCAT-DTG-AF mitochondria from *n* = 2 mice). Data are shown as mean ± SEM, *P < 0.05, **P < 0.01, ***P < 0.001.

While arrhythmias may alter the hemodynamic output from the heart, the ability of the macrovascular and microvascular circulation to autoregulate is an important homeostatic mechanism that may compensate for changes in pressure (26). Cerebral vessels are known to autoregulate to maintain flow to the brain (26, 27). We asked whether AF results in changes in microvasculature, such as resistance arteries, that may play a role in cognitive decline. We performed histology on mesenteric resistance arteries as well as brain pial arteries stained for elastin from DTG-AF and control mice. We found mesenteric and brain pial arteries in DTG-AF mice demonstrated ~1.5× thicker walls than control arteries (Figure 3, A–D) (mesenteric: 26.1 ± 4.2 versus 38.4 ± 3.3 μm, brain pial: 10.0 ± 0.7 versus 16.3 ± 1.6 μm). Wall thickness was predominated by the smooth muscle–containing tunica media. We also noted substantial heterogeneity in the thickness of DTG-AF mesenteric arteries, suggesting that AF may lead to variable remodeling of the arteries, perhaps due to variations in hemodynamic forces experienced during AF. In order to assess the structural basis of vascular alterations in DTG-AF mice, we performed transmission electron microscopy on isolated mesenteric arteries and brain pial arteries. The architecture of DTG-AF mesenteric arteries showed typical internal elastic lamina and multiple layers of smooth muscle cells akin to control (Figure 4A). Pial arteries showed similarly preserved structure with endothelial cells forming the tunica intima and smooth muscles cells in the tunica media without disruption of macrostructure (Figure 4D). Higher-magnification analysis also demonstrated preserved actin filaments and dense bodies within smooth muscle cells. However, mitochondria throughout the vessel walls in both pial and mesenteric arteries were of significantly larger size than controls (Figure 4, B, C, E, and F) (mesenteric: 119,703 ± 7,387 versus 149,829 ± 5,578 nm^2^, brain pial: 126,396 ± 6,931 versus 173,951 ± 14,602 nm^2^). We have previously demonstrated mitochondrial dysfunction in the cardiomyocytes in DTG-AF mice (11, 28), and our results here suggest further mitochondrial involvement in vasculature. Coexpression of mCAT in DTG-AF mice (mCAT-DTG-AF mice) in order to reduce ROS significantly decreased mitochondrial size in mesenteric arteries and, in pial arteries, resulted in mitochondria nonsignificantly different from control (Figure 4, B, C, E, and F) (mesenteric mCAT-DTG-AF: 90,083 ± 5,658 nm^2^, brain pial: 140,941 ± 16,467 nm^2^). Notably, the mutant NaV1.5 channel in DTG-AF mice is selectively expressed in the heart (9), suggesting that mitochondrial dysfunction in the vessels arises secondary to systemic effects of AF. We next asked whether these organelle- and tissue-level structural alterations in DTG-AF arteries lead to vascular functional changes.

### Loss of myogenic tone in arteries from DTG-AF mice.

**Figure 5:**
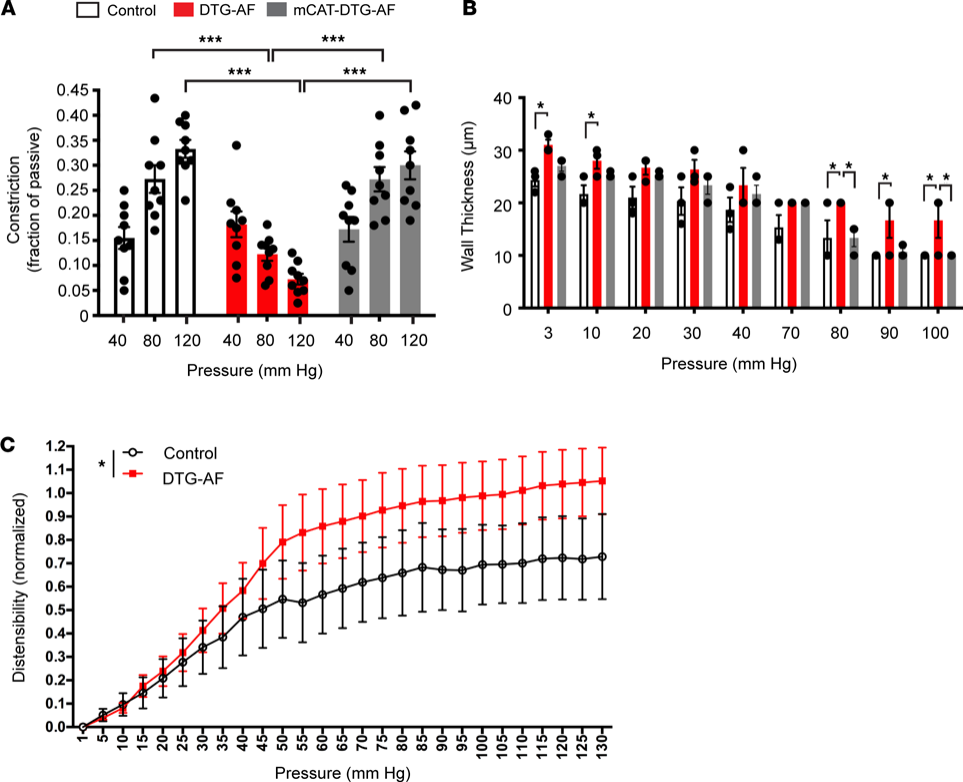
Loss of myogenic tone and increased arterial wall thickness in DTG-AF mice correctable with mCAT. (**A**) Myogenic tone of third-order mesenteric arteries measured ex vivo as fraction of active diameter to maximal passive diameter in the absence of Ca^2+^, at each intraluminal pressure. Control mice demonstrate typical myogenic response of increased constriction with increased intraluminal pressure. DTG-AF mice have significantly reduced constriction at 80 mmHg and 120 mmHg, which is prevented in mCAT-DTG-AF mice. Two-way repeated-measures ANOVA with Tukey’s multiple-comparison test: 80 mmHg control versus DTG-AF *P* < 0.001, DTG-AF versus mCAT-DTG-AF *P* < 0.001, mCAT-DTG-AF versus control *P* = NS, 120 mm Hg control versus DTG-AF *P* < 0.001, DTG-AF versus mCAT-DTG-AF *P* < 0.001, mCAT-DTG-AF versus control *P* = NS (*n* = 9 control, *n* = 9 DTG-AF, *n* = 9 mCAT-DTG-AF mesenteric arteries). (**B**) Wall thickness of mesenteric arteries at increasing intraluminal pressures. DTG-AF vessels are significantly thicker than control vessels, particularly at low and high ends of pressure, and mCAT coexpression normalizes wall thickness. Two-way repeated-measures ANOVA with Tukey’s multiple-comparison test, all significant comparisons denoted with *P < 0.05 (*n* = 3 control, *n* = 3 DTG-AF, *n* = 3 mCAT-DTG-AF vessels). (**C**) Distensibility studies via measuring vessel diameter under varying pressures in Ca^2+^ free solution reveals significantly differing effect of pressure on distensibility in DTG-AF mice. Generalized estimating equations (*P* < 0.05) (*n* = 4 control and *n* = 7 DTG-AF vessels). Data are shown as mean ± SEM, *P < 0.05, ***P < 0.001.

We isolated mesenteric resistance arteries from 3-month-old controls and DTG-AF mice and performed ex vivo pressure response studies as previously described (29). In response to increasing intraluminal pressures of 40, 80, and 120 mmHg, control arteries exhibited a typical myogenic tone response and constriction (Figure 5A). However, DTG-AF arteries did not show this myogenic response, and the level of constriction at 80 mmHg and 120 mmHg was significantly lower than control arteries (Figure 5A) (80 mmHg: 0.27 ± 0.03 versus 0.12 ± 0.01, 120 mmHg: 0.33 ± 0.02 versus 0.07 ± 0.01). We additionally assessed the wall thickness of arteries at varying pressures in a passive state in the absence of Ca^2+^. DTG-AF mice consistently demonstrated thicker arterial walls than controls, most significant at low and high pressures (Figure 5B). Reduced constriction in DTG-AF mice may be due to dysfunction in constriction of thickened artery, such as reduction in vascular smooth muscle cell contractility. DTG-AF mesenteric arteries also demonstrate altered response to increasing pressure in a passive state, measured as increased distensibility than control arteries (Figure 5C), suggesting changes in mechanical properties.

The mitochondrial enlargement in DTG-AF vessels seen on electron microscopy was suggestive of increased oxidative stress that leads to mitochondrial dysfunction. We have previously demonstrated mitochondrial derangements in cardiomyocytes of DTG-AF mice and shown that expression of mCAT reduced ROS and significantly reduced AF burden and atrial remodeling (11, 28). We asked whether attenuating mitochondrial oxidative stress via coexpression of mCAT (mCAT-DTG-AF mice) would be able to prevent the vascular dysfunction seen in DTG-AF mice. We demonstrated that expression of mCAT prevented the loss of myogenic tone (Figure 5A) (mCAT-DTG-AF: 80 mmHg, 0.27 ± 0.02; 120 mmHg, 0.30 ± 0.03). Furthermore, wall thickness changes were also prevented with mCAT coexpression (Figure 5B). These results suggest that AF results in vascular remodeling with mitochondrial dysfunction, increased wall thickness and distensibility, and deficient myogenic autoregulation. However, attenuation of mitochondrial oxidative stress is sufficient to prevent vascular dysfunction and maintain normal myogenic tone.

### mCAT expression improves learning and cognitive flexibility in DTG-AF mice.

**Figure 6:**
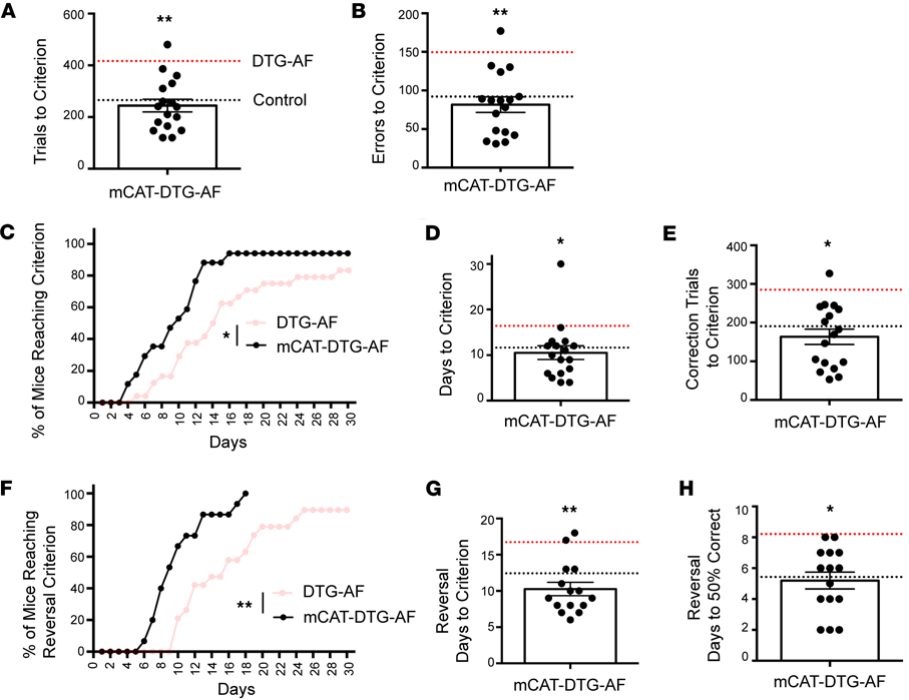
Impaired visual learning and cognitive flexibility in DTG-AF mice rescuable by mCAT. Data from Figure 1 visualized as dashed lines depicting the means of control and DTG-AF groups alongside mCAT-DTG-AF mice. Asterisks represent comparison between mCAT-DTG-AF and DTG-AF groups. (**A**) Expression of mCAT results in significantly improved performance in trials to criterion than DTG-AF with no difference from control. One-way ANOVA and Tukey’s test: DTG-AF versus mCAT-DTG-AF, *P* < 0.01; mCAT-DTG-AF versus control, *P* = NS) (*n* = 15 control, *n* = 24 DTG-AF, *n* = 17 mCAT-DTG-AF mice). (**B**) mCAT expression reduces the number of errors made by DTG-AF mice. (**C**) Percentage of mice reaching criterion at each day with mCAT-DTG-AF mice performing significantly better than DTG-AF mice (log-rank Mantel-Cox test, χ^2^ = 6.23, *P* = 0.01) and nonsignificantly different than control (χ^2^ = 0.43, *P* = 0.51) (*P < 0.02 per α corrected for multiple comparisons with Bonferroni’s). DTG-AF data from Figure 1 translucent. (**D**) mCAT expression improves days required to reach criterion compared with DTG-AF. (**E**) Correction trials to criterion demonstrate a similar pattern. (**F**) Percentage of mice reaching criterion each day after the correct stimulus is reversed. mCAT coexpression significantly improves number of mice reaching criterion each day compared with DTG-AF. Log-rank Mantel-Cox (DTG-AF versus mCAT-DTG-AF χ^2^ = 13.30, *P* <0.001) (mCAT-DTG-AF versus control χ^2^ = 2.18, *P* = 0.14) (*n* = 14 control, *n* = 19 DTG-AF, *n* = 15 mCAT-DTG-AF) (**P < 0.003 per α corrected for multiple comparisons with Bonferroni’s test). (**G**) mCAT coexpression significantly reduces days required to learn the reversed criterion. (**H**) Analysis of early reversal shows mCAT-DTG-AF mice unlearn the original criterion and reach 50% chance performance faster than DTG-AF and same as control mice. Data are shown as mean ± SEM, *P < 0.05, **P < 0.01, except as noted in **F**.

We further sought to determine whether reducing mitochondrial ROS by expression of mCAT would attenuate the development of cognitive dysfunction. We performed touch screen behavioral testing on triple transgenic mCAT-DTG-AF mice and compared against DTG-AF and control mice from Figure 1. In line with our prior study, mCAT-DTG-AF mice have significantly reduced AF burden compared with DTG-AF mice (Supplemental Figure 3). We found mCAT-DTG-AF mice required a significantly fewer number of trials to reach criterion than DTG-AF mice and achieved the same performance as controls (Figure 6A) (mCAT-DTG-AF 244 ± 25, DTG-AF [from Figure 1] 417 ± 40, Control [from Figure 1] 265 ± 30). mCAT-DTG-AF mice also made 45% fewer errors during learning than DTG-AF mice (Figure 6B) (mCAT-DTG-AF 82 ± 10, DTG-AF 150 ± 17, Control 92 ± 12). Survival analysis of percentage of mice reaching criterion shows mCAT-DTG-AF mice reached criterion faster than DTG-AF mice in 25% fewer days (Figure 6, C and D) (mCAT-DTG-AF 10.5 ± 1.5, DTG-AF 16.4 ± 1.7, Control 11.6 ± 1.7). The improved performance of mCAT-DTG-AF mice was also evident in requiring fewer correction trials (Figure 6E) (mCAT-DTG-AF 163 ± 20, DTG-AF 285 ± 32, Control 190 ± 28). On additional metrics, mCAT-DTG-AF mice made significantly fewer inactive touches than DTG-AF mice but did not show any differences in perseveration index or reward collection latency (Supplemental Figure 4, A–C). We also performed reversal learning on mCAT-DTG-AF mice, and we found improved cognitive flexibility compared with DTG-AF mice and on par with controls mice, based on percentage of mice reaching reversal criterion per day (Figure 6F), total days to criterion (Figure 6G) (mCAT-DTG-AF 10.3 ± 0.9, DTG-AF 16.7 ± 1.5, Control 12.4 ± 0.9), total trials to criterion (Supplemental Figure 5A), a trend in total errors to criterion (Supplemental Figure 5B), and total correction trials to criterion (Supplemental Figure 5C). On early reversal analysis, mCAT-DTG-AF mice also took 0.6× fewer days to reach 50% chance and unlearn the original stimulus than DTG-AF mice (Figure 6H) (mCAT-DTG-AF 5.2 ± 0.5, DTG-AF 8.2 ± 1.0, Control 5.4 ± 0.6). Taken together, these data show that cognitive deficits in the visual learning domain of our mouse model of AF can be prevented by attenuating mitochondrial oxidative stress. These results were also consistent on subanalysis of a narrower age range of mice 2–4 months old (Supplemental Figure 6). We also performed subanalysis of male versus female mice for sex differences and found that male DTG-AF predominantly showed cognitive decline in touch screen testing and required significantly greater trials to reach criterion and made more errors than female DTG-AF mice (Supplemental Figure 7, A–I). We implanted s.c. ECG telemeters in male and female DTG-AF mice and found that male mice demonstrated a 3-fold higher burden of AF (Supplemental Figure 7J), which may explain this difference in cognitive performance.

We further tested the performance of DTG-AF and mCAT-DTG-AF mice in another behavioral assay utilizing the open field test, where mice were placed in an empty, walled chamber and their movement was monitored to measure exploratory behavior and locomotor activity (30, 31) (Supplemental Figure 8A). DTG-AF mice did not show significantly different center time or vertical movements than controls mice (Supplemental Figure 8,C and D), suggesting that AF burden did not affect exploratory behavior or emotional anxiety in these mice. However, in a subgroup analysis of mice older than 6 months, the DTG-AF group demonstrated significantly greater vertical movements than controls, which was prevented in the mCAT-DTG-AF group (Supplemental Figure 9, A–C). This suggests that aging may lead DTG-AF mice to lose the self-preservation instinct to limit exploration of an unfamiliar environment, and such behavior can be prevented by reducing mitochondrial oxidative stress.

### Reduced cerebral perfusion in DTG-AF mice is restored via mCAT expression or ranolazine treatment.

**Figure 7:**
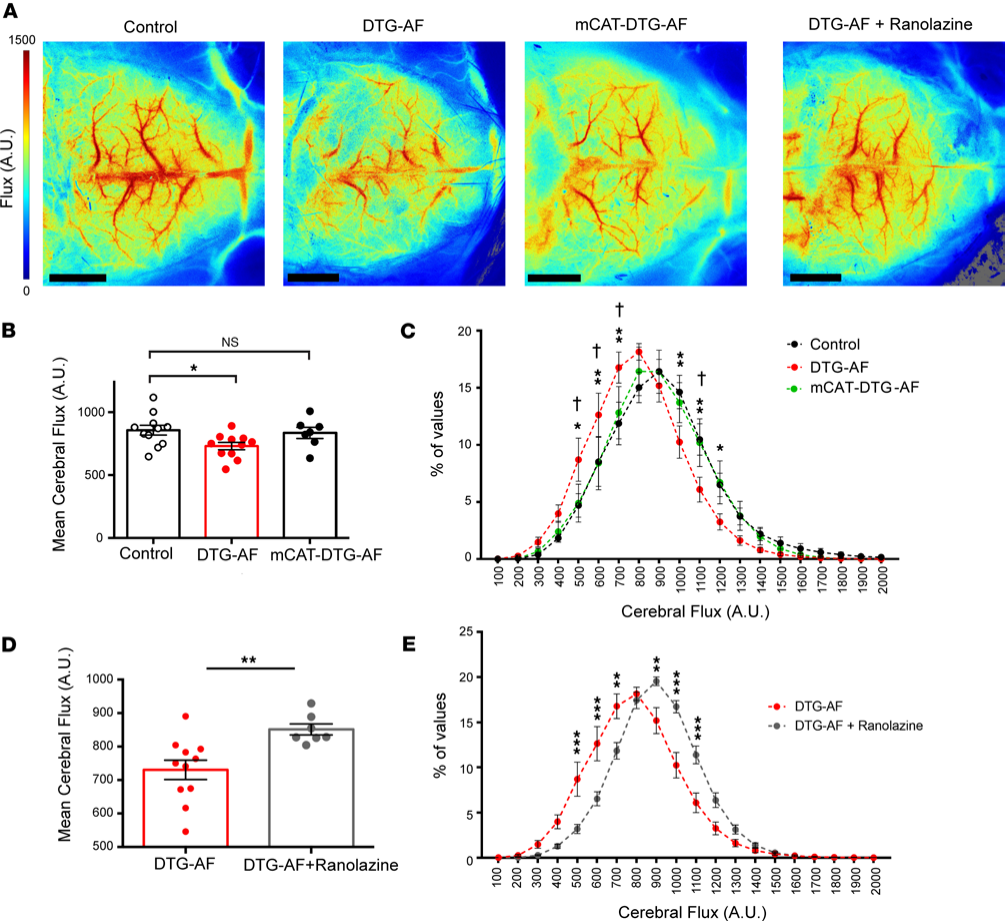
Reduced cerebral perfusion in DTG-AF mice is restored via mCAT expression or ranolazine treatment. (**A**) Representative images from laser speckle contrast imaging of the cerebrum in control (*n* = 11 mice), DTG-AF (*n* = 11), mCAT-DTG-AF (*n* = 7), and DTG-AF mice treated with ranolazine (*n* = 7). (**B**) Quantification of mean flux across the cerebrum. DTG-AF mice demonstrate significantly reduced perfusion in the cerebrum compared with controls, while mCAT-DTG-AF mice show normalized perfusion no different from controls (1-way ANOVA with Tukey’s test: control versus DTG-AF *P* < 0.05, control versus mCAT-DTG-AF *P* = NS). (**C**) Distribution of flux values reveals leftward shift in DTG-AF brains with greater area of cerebrum receiving lower perfusion (2-way ANOVA with Tukey’s multiple-comparison test). Asterisks denote control versus DTG-AF (*P < 0.05, **P < 0.01) while daggers denote DTG-AF versus mCAT-DTG-AF (^†^*P* < 0.05); all control versus mCAT-DTG-AF comparisons *P* = NS. (**D**) DTG-AF mice treated with ranolazine injection to convert to sinus rhythm demonstrate significantly improved brain perfusion (unpaired 2-tailed *t* test, **P < 0.01). (**E**) Distribution of flux values reveals correction of leftward shift in perfusion in DTG-AF mice treated with ranolazine (2-way ANOVA with Šídák’s test, **P < 0.01, ***P < 0.001). Data are shown as mean ± SEM.

Our data suggest that DTG-AF mice demonstrate vascular dysfunction and reduced cognitive performance that are improved by mCAT expression. To determine if mCAT expression is indeed improving cognitive performance via reducing AF burden and vascular dysfunction, we investigated brain perfusion via laser speckle contrast imaging (Figure 7A). We found that DTG-AF mice had significantly reduced cerebral perfusion compared with controls, while mCAT-DTG-AF showed perfusion no different from controls (Figure 7B) (Control 857 ± 39, DTG-AF 730 ± 29, mCAT-DTG-AF 836 ± 44 flux units). The distribution of flux values in DTG-AF mice were leftward shifted, with greater areas of cerebrum receiving lower perfusion, which was corrected with mCAT expression (Figure 7C). Importantly, DTG-AF mice injected with ranolazine to pharmacologically convert to sinus rhythm showed significantly improved brain perfusion compared with the untreated DTG-AF mice (Figure 7D) (DTG-AF + Ranolazine 851 ± 16 versus DTG-AF 730 ± 29 flux units). The distribution of flux values in DTG-AF mice cardioverted with ranolazine showed a correction of the leftward shift in perfusion of untreated DTG-AF mice (Figure 7E). Since it is possible that the slightly reduced LVEF may be a contributing factor to cognitive dysfunction, we cannot definitively link causality between AF and cognitive dysfunction. However, we performed further testing using echocardiography to assess DTG-AF heart function in atrial fibrillation and after injection with ranolazine to cardiovert the mice to normal sinus rhythm. We found that there were no significant differences in the heart function, which was slightly reduced (Supplemental Figure 10).

## Discussion

There has been increasing concerns and research confirming that patients with AF, despite being on anticoagulation, are at high risk for developing neurological deficits (2, 32). However, the mechanism behind the effect of heart rhythm on the brain is poorly understood. Investigations into cognitive decline and dementia due to AF are challenging due to (a) need for long-term followup for detection and assessment; (b) lack of tests to diagnose and understand its progression over time; and finally, (c) a lack of an animal model for investigation.

There are multiple theorized mechanisms by which AF may increase the risk of neurological decline in patients. A leading hypothesis is that this decline results from asymptomatic SCIs that can only be detected on imaging. While SCIs can be found in healthy individuals, patients with AF have a higher prevalence of SCIs as well as a greater number of infarcts (17, 18). Furthermore, patients with persistent AF (persAF) showed greater SCIs than patients with paroxysmal AF (pAF), suggesting an arrhythmia burden dose response (17). AF is also associated with systemic inflammation and upregulation of signaling factors such as C-reactive protein, IL-6, and TNF-α that may exert effects on the brain (32). Endothelial dysfunction has also been recognized in AF, although it’s role as an upstream instigator or downstream pathology is unknown (33, 34). Previous studies have focused on AF as a result of vascular impairment (35), whereas we propose a paradigm shift, namely that AF is the cause of vascular dysfunction leading to cognitive impairment.

Insight into the mechanistic effects of AF on the brain has been previously limited by the lack of studies in animal models with persAF. Furthermore, human clinical trials require an extensive follow-up duration and testing to assess for development of cognitive deficits. Previous studies of cardiovascular causes of cognitive decline have primarily utilized models of global hypoperfusion, such as stenosis via bilateral carotid artery coils in rodents (36, 37). However, to our knowledge, there have been no investigations of cognitive decline in animal models due to cardiac arrhythmia.

Herein, we show that our DTG-AF mouse model with spontaneous, prolonged AF episodes due to a cardiac specific altered transgene, displayed neurocognitive dysfunction, and caused behavioral alterations, namely reduced visual learning, reduced cognitive flexibility, and loss of typical environmental anxiety. To our knowledge, this is the first demonstration that altered cardiac rhythm can result in neurobehavioral consequences in a mouse model. While the DTG-AF mice did not show infarcts on brain MRI, they demonstrated ventriculomegaly and increased diffusion in the left frontal lobe. Furthermore, the microcirculation resistance arteries of DTG-AF mice show loss of myogenic tone, increased wall thickness, and mitochondrial enlargement. Importantly, both the neurocognitive and vascular dysfunction seen in DTG-AF mice is prevented by expression of mCAT to reduce mitochondrial ROS. It is possible that the slightly reduced LVEF and cardiomyopathy of the DTG-AF may be a causal factor in the development of cognitive dysfunction.

Cognitive tests in patients with AF have demonstrated that deficits occur in the particular domain of visual-spatial and visuo-spatial executive function (17, 38). The touch screen behavioral test is an assessment of visual processing involving the ability to discriminate between 2 environmental stimuli, learn stimulus-reward relationships, and during reversal learning, inhibit a previously formed association and begin relearning. The impairments seen in DTG-AF mice in this test fit well with the visual-spatial impairments seen in patients. Prior studies in rodents have localized acquisition learning in the touch screen task to require the dorsolateral striatum, and reversal learning to require the orbitofrontal cortex and dorsolateral striatum (16, 39, 40). While brain MRI did not reveal infarcts in these regions of the brain in DTG-AF mice, we did observe an increase in diffusion in the left frontal lobe that could be consistent with reduced cellularity or vasogenic edema that could impair function. The enlarged lateral ventricles in DTG-AF mice could also represent brain atrophy due to cell loss or abnormal cerebrospinal fluid accumulation that may be due to reduced venous drainage (41).

Cerebral autoregulation relies on the myogenic tone of arteries to constrict during high pressures and dilate during low pressures to regulate cerebral blood flow (26). If intraluminal pressure exceeds the ability of the artery to constrict, autoregulatory breakthrough can occur, resulting in vessel dilation, large increase in blood flow, and vasogenic edema; this process is well studied in hypertensive encephalopathy (27, 42). AF has been shown to reduce cerebral blood flow in patients (43). In our AF murine model, there is a loss of myogenic tone, which could result in a greater susceptibility to autoregulatory breakthrough episodes subjecting the brain to edema. This would fit with our observation of increased ADC suggestive of vasogenic edema in the left frontal lobe. Indeed, patients with AF have been shown to have impaired cerebral autoregulation on squat-to-stand testing, with greater transmission of mean arterial pressure (MAP) to the brain (44). In clinical practice, many patients with AF commonly have comorbid hypertension, and the loss of myogenic tone could increase the risk of hypertensive encephalopathy and contribute to cognitive decline. Our DTG-AF mouse model has the potential to be combined with other stressors such as inducible hypertension to study the effects on the brain and cognitive decline (45).

Interestingly, we found that expression of mCAT is sufficient to reverse the neurocognitive dysfunction seen in DTG-AF mice, in both acquisition and reversal learning in the touch screen test, as well as environmental anxiety in the open field test. Given our prior work demonstrating that mCAT expression is able to significantly reduce the AF burden in our DTG-AF mice, these results suggest that controlling rhythm may also offer cognitive protection. Further studies are necessary to evaluate whether the correction of myogenic tone in mCAT-DTG-AF mice is due to primary effects on the vasculature or due to secondary effects caused by reduction of arrhythmia burden and systemically propagated factors. mCAT expression does not completely eliminate AF burden however appears to fully restore myogenic tone as well as normal cognitive function in touch screen tests. This suggests that mCAT may be working downstream of AF and in the vasculature, and that mitochondrial oxidative stress may play an important role in the pathophysiology.

We recognize that mCAT expression was not specific to the cardiovascular system, and therefore, it is possible that it plays a role in the brain. Prior work has found that specific expression of mCAT in neurons versus astrocytes has differing effects, with neuronal expression increasing center time and reducing anxiety in the open field test (46), while astrocytic expression reduces center time and increases anxiety (47). Whole-mouse expression of mCAT has been previously reported to have no effect on open field performance (48). In the context of these results, it appears that the restoration of open field anxiety we observed in mCAT-DTG-AF mice likely arises from the action of mCAT in reducing AF burden.

We have previously shown that DTG-AF mice demonstrate cardiomyopathy that is likely multifactorial from tachycardia-induced cardiomyopathy as well as contractility defects from the genetic mutation. It is possible that cardiomyopathy also leads to cognitive decline, although DTG-AF mice showed only a slight reduction in ejection fraction, and we show that reversing AF via administration of ranolazine does not change the ejection fraction.

In summary, this is the first report to our knowledge of an animal model of AF with cognitive dysfunction that models similarly the deleterious cognitive effects of AF seen in humans. We demonstrate AF-induced vascular remodeling with comprehensive investigation of vascular structure, ultrastructure, and functional dynamics. This model will be invaluable to further studies to understand the mechanisms in which AF affects microcirculation and macrocirculation of the neurovascular system, as well as mitochondrial changes in the vasculature. It may also be a useful model to further elucidate and test therapeutic targets to treat AF as well the deleterious effects of such abnormal irregular rhythm on brain function, which has otherwise been poorly understood. Improved understanding of the mechanism of cognitive effects of AF would have significant implications on clinical management such as rate versus rhythm control and further target therapeutics in addition to the current recommendations of systemic anticoagulation.

## Methods

### General experimental approaches

All experimental procedures and analyses were performed in a blinded fashion. No data points, samples, or mice were excluded from the study.

### Sex as a biological variable

Male and female mice were analyzed in this study. Please see Supplemental Figures 7 and 8 for analysis of sex differences.

### Animal subjects

The mouse model used in this study was previously generated (9–11). In brief, mice expressing the human sodium channel *SCN5A* with an Ala-substitution at position F1759 under a modified heart-specific α-MHC promoter with tetracycline inducibility were crossed with cardiac-specific reverse tetracycline-controlled trans-activator protein (rtTA) mice (from Mutant Mouse Resource and Research Center). Possibly blind mice with both copies of the rd1 recessive mutation from FVB/N background were not used in the study groups. Homozygous mCAT mice (stock no. 016197, Jackson Laboratory) was used to generate F1759A/rtTA/mCAT mice (mCAT-DTG-AF). Male and female mice 8 weeks to 12 months of age were used. Single transgenic F1759A or genotype negative littermates were used as controls. All mice were group-housed by sex in a temperature and humidity-controlled vivarium at Columbia University Medical Center and maintained on a standard 12-hour light/dark cycle (lights on at 7 am) with food and water provided ad libitum (except for the touch screen experiment, see below). Behavioral testing was performed in littermates. All behavioral experiments were performed in the Columbia Mouse Neurobehavior Core facility during the light phase (between 10 am and 4 pm). All tests were conducted in accordance with national guidelines (National Institutes of Health) and approved by the Institutional Animal Care and Use Committee of Columbia University.

### Immunoblots

Cardiomyocytes and brain tissue were homogenized in a 1% Triton X-100 buffer containing (in mM): 50 Tris-HCl (pH 7.4), 150 NaCl, 10 EDTA, 10 EGTA, and protease inhibitors. The lysates were incubated on ice for 30 minutes and centrifuged at 20,800*g* at 4°C for 10 minutes. Supernatants were collected. Proteins were size-fractionated on SDS-PAGE, transferred to nitrocellulose membranes, and probed with anti-FLAG (catalog A8592, Sigma-Aldrich), anti-NaV1.5 (catalog ASC-005, Alomone) and anti-tubulin (catalog sc-12462-R, Santa Cruz Biotechnology Inc.) antibodies. Detection and quantification were performed with a CCD camera (Carestream) and ImageQuant software (GE Healthcare), respectively.

### Behavioral tests

#### Touch screen test.

Pairwise visual discrimination and reversal learning were tested in the automated Bussey-Saksida touch screen equipment for mice (Campden Instruments Ltd/Lafayette Instruments), following methods described previously (12, 13, 39, 49–52). A palatable liquid nutritional supplement (Strawberry Ensure Plus) diluted to 50% with water was used as the reinforcer. The volume of each reinforcement was 20 μL. Before pretraining subject mice were weighed and placed on a restricted diet of 2–4 g of rodent chow per mouse per day. Body weight was carefully monitored throughout the acquisition and reversal training, to ensure that a minimum of 85% of free feeding body weight was maintained for each mouse.

Pretraining was performed as described in detail previously (15). Briefly, mice advance through 5 stages in pretraining. Stage 1: habituation to the chamber without touch screen stimuli. Stage 2: presentation of stimuli on a single window with triple the reinforcer volume on successful touch. Stage 3: image remains presented on screen until touched. Stage 4: next trial initiated upon mouse entering food magazine. Stage 5: touching of blank touch screen results in time-out. After pretraining was successfully completed mice were advanced to the pairwise visual discrimination test where 2 different visual stimuli, a spider and an airplane, are presented in a spatially pseudorandomized manner on the 2 touch screen windows (15). Selection of the correct image (determination of correct image was balanced across mice per genotype) results in reinforcer reward while incorrect responses lead to a correction trial where stimuli are represented, and correction trials repeated until a correct response is made. Mice underwent sessions of 30 trials with 15-second intertrial intervals. Successful learning was defined as achieving a criterion of average ≥ 80% correct responses in 2 consecutive days with 30 complete trials.

Animals that failed to reach criterion during the acquisition phase were not advanced onto the reversal phase. Reversal training was initiated approximately 3 days after the last day of acquisition learning. Correct stimulus response was reversed for each mouse, with the same criterion of average of ≥ 80% correct responses on 2 consecutive days. A 30-day cutoff was used for both acquisition and reversal, and animals that failed to reach criterion in 30 days were given a number “30” as their days to reach criterion during data analysis. Number of days, total trials, total errors, and total correction trials to criterion as well as percentage of mice reaching criterion per day, inactive touches, perseveration index, and reward collection latency were compared between genotypes. Perseveration index was defined as number of correction trials/number of errors. Three mice that did not achieve 80% correct responses were excluded from results.

#### Open field test.

The open field test was performed following previously described protocols (53, 54). Exploration was monitored during a 60-minute session with Activity Monitor Version 7 tracking software (Med Associates). Each mouse was placed in the center of a clear Plexiglas arena (27.31 × 27.31 × 20.32 cm, Med Associates ENV-510) lit with dim light (~5 lux), and allowed to ambulate freely. Infrared (IR) beams embedded along the *x*, *y*, and *z* axes of the arena automatically track distance moved, vertical movements, and time spent in center zone (14.29 × 14.29 cm). Data are analyzed in 6, 10-minute time bins. Arenas were cleaned with 70% ethanol and thoroughly dried between trials.

### Magnetic resonance imaging

MRI was performed on a Bruker BioSpec 9.4T Magnetic Resonance Imager. The mice were anesthetized with 1%–2% isoflurane mixed with medical air via a nose cone. The concentration of the isoflurane was adjusted during the procedure to maintain the respiration rate in the range of 40–70 breaths/min using a respiration pillow attached to a monitoring system (SA Instruments). Body temperature was maintained around 37°C using a flowing water heating pad. A 23-mm 1H circularly polarized transmit/receive capable mouse head volume coil was used for the imaging. After low-resolution T1-weighted scout images were obtained initially for localization of the brain, a T2 rapid acquisition with relaxation enhancement (RARE) sequence was used with the following parameters: repetition time (TR) = 3,000 ms, echo time (TE) = 43.5 ms, field of view (FOV) = 20 × 15 mm, matrix size = 284 × 214, slice thickness = 0.5 mm with 16 slices to cover the entire brain in the coronal direction. The 3D segmentation and volumes of the ventricle and whole brain were analyzed with Analyze 14 software. For diffusion-weighted images, the parameters were TR = 2800 ms, TE = 20 ms, gradient duration = 2.5 ms, gradient separation = 8.5 ms, and 4 b-values of 200, 450, 650, and 1,000 s/mm². The ADC were analyzed with Bruker Paravision 6.0.1 using the Image Sequence Analysis tool. For perfusion images, T2-weighted contrast-enhanced images were acquired, and T2-dynamic images were acquired before and 12 minutes after intraperitoneal injections of Gadodiamide (Omniscan; GE Healthcare) at a dosage of 10 mmol/kg, with the parameters described above for T2 images for volume measurement except FOV = 18 × 15 mm, matrix size = 225 × 174, slice thickness = 0.36 mm, with 22 slices, and a repetition of 15 for the dynamic images. Cerebral blood volume (CBV) ratio was analyzed with the method described previously (55).

### Vascular myogenic tone studies

Vascular myogenic tone was measured as previously described (29). Briefly, third-order mesenteric arteries were dissected and placed in a petri dish with ice-cold physiologic saline solution (in mM, 142 NaCl, 4.7 KCl, 3.5 MgSO_4_, 1.2 KH_2_PO_4_, 2.5 CaCl_2_, 10 glucose, 10 HEPES, pH adjusted to 7.4 with NaOH at 10°C). The vessels were mounted onto 2 glass cannulas in a vessel chamber (Living Systems Instrumentation) and equilibrated at 50 mm Hg for 20 minutes in a Krebs buffer. The viability of the vessel preparations was then briefly assessed using 20–40 mM KCl followed by a thorough washout. Vessels that failed to constrict were deemed nonviable and were discarded. Diameter changes in pressurized segments were measured at 40, 80, and 120 mm Hg using automatic edge detection (Living Systems). Vessels were first subjected to a stepwise increase in intraluminal pressure from 40–120 mm Hg followed by a stepwise decrease in intraluminal pressure from 120 to 40 mm Hg. The vessel diameter was monitored continuously for at least 5 minutes, to ensure a steady state was reached, at each pressure step. The arteries were then incubated in a Ca^2+^-free Krebs solution in order to prevent contraction, and the pressure-diameter measurements were repeated. The degree of myogenic tone was calculated as (Ca^2+^-free diameter – Ca^2+^-containing diameter)/(Ca^2+^-free diameter) × 100. Wall thickness was calculated as (outer diameter of the vessel – the inner diameter of the vessel/2) in the Ca^2+^-free solution at various intramural pressures. Distensibility was evaluated and calculated as previously described (29).

### Histopathology

Mesenteric arteries and brain pial arteries were fixed in 10% formalin and embedded in paraffin. Cross sections were stained with hematoxylin and eosin (H&E) and elastin Van Gieson stain and imaged under light microscopy at 60× magnification. Average wall thickness was measured in ImageJ (NIH) by segmentation of the vessel wall by tracing the tunica intima and adventitial layers. The mean thickness of the resulting segmented vessel wall was determined utilizing the Local Thickness plugin in ImageJ, which calculates the thickness at each point circumferentially of the vessel wall and can be averaged around the entire vessel to determine mean thickness.

### Transmission electron microscopy

Freshly dissected third order mesenteric and brain pial arteries were fixed in a solution of 2% paraformaldehyde and 2.5% glutaraldehyde in 0.1 M sodium cacodylate at 4**°**C for 24 hours. Samples were then treated with 1% osmium tetroxide in 100 mM cacodylate buffer pH 7.4 for 1 hour, washed in distilled water 4 times (10 min/wash), and then treated with 2% aqueous uranyl acetate overnight at 4°C in the dark. Samples were then washed and sequentially dehydrated with increasing concentrations of ethanol (20, 30, 50, 70, 90, and 100%) for 30 minutes each, followed by 3 additional treatments with 100% ethanol for 20 minutes each. Samples were then infiltrated with increasing concentrations of Spurr’s resin (25% for 1 hour, 50% for 1 hour, 75% for 1 hour, 100% for 1 hour, 100% overnight at room temperature) and then incubated overnight at 70°C in a resin mold. Toluidine blue stained sections (1 μm thick) were cut initially to localize the arteries before cutting adjacent 80 nm thick sections using the Leica ultramicrotome with a diamond knife. Grids with sections were stained with 4% uranyl acetate for 5 minutes, washed, and then finally stained with lead citrate to enhance contrast. Imaging then took place using a ThermoFisher Talos L120C transmission electron microscope operating at 120 kV. Mitochondrial size was quantified by manual segmentation of individual mitochondria in ImageJ.

### Laser speckle contrast brain imaging

Brain perfusion was assessed using laser speckle contrast imaging. Mice were anesthetized with inhaled isoflurane and a midline scalp incision made to reflect skin and expose the cerebrum covered by intact transparent cranium. Phosphate-buffered saline warmed to 37°C was applied to the skull to prevent drying. Images were acquired with a moorFLPI-2 laser speckle contrast imager (Moor Instruments Inc.) at a frame interval of every 5 seconds. Speckle contrast images were analyzed using MoorFLPI-2 Review software V5.0 with a region of interest placed over the cerebrum to quantify mean flux and generate histograms of flux distribution.

For pharmacological cardioversion of arrhythmia, DTG-AF mice underwent s.c. 4-lead ECG as previously described (9–11) and were confirmed to demonstrate AF defined as absence of P waves and irregular R-R intervals. Mice were then injected intraperitoneally with 20 mg/kg of ranolazine and ECG repeated 2–3 hours after treatment to confirm conversion to sinus rhythm before undergoing immediate laser speckle imaging.

### Telemetry and ECG analysis

The s.c. 3-lead ECGs of isoflurane-anesthetized mice were performed using EMKA ECG and recorded using Iox. QT intervals were measured manually using Ponemah 3 software. Telemetry devices (Data Sciences International, model ETA-F10) were implanted in 3-month-old mice. Recordings were started 1 week after implantation. AF was defined as absence of P waves and irregular R-R intervals for more than 1 second.

### Transthoracic echocardiography

Echocardiography was performed on anesthetized mice using a VisualSonics Vevo 2100 high-resolution imaging system with a 30 mHz imaging transducer. Left ventricular end diastolic volume (LVEDV) and left ventricular end systolic volume (LVESV) were measured in parasternal short axis views and 4 chamber views from which LVEF was then calculated using Simpson’s formula.

### Statistics

For open field test data, 2-way repeated-measures ANOVA, with time as the within-subject factor and genotype as the between-patient factor, was performed on distance traveled, center time, and vertical movements with post hoc Tukey’s multiple-comparison test. Survival data of the touch screen test were analyzed using log-rank Mantel-Cox test, with Bonferroni’s correction for multiple comparisons with 3 groups. Other variables in the touch screen test were analyzed with unpaired 2-tailed *t* test for 2 groups and 1-way ANOVA and Tukey’s post hoc for 3 groups. MRI data were analyzed via unpaired *t* test and vascular tone data via 2-way repeated-measures ANOVA with Tukey’s post hoc test. Analyses were performed in GraphPad Prism 6, and statistical significance was defined as *P* < 0.05. Distensibility was analyzed via generalized estimating equations utilizing SPSS software. Data presented as mean ± SEM.

### Study approval

The IACUC at Columbia University approved all animal experiments.

### Data availability

Values for all data points in graphs are reported in the Supporting Data Values file.

## Author contributions

EYW conceptualized the study. EYW, PG, RJ, NS, UMRA, DZS, AF, EL, ADD, SP, PD, CS, YS, CNG, SRR performed experiments and data analysis. PG and EYW wrote the first full draft of the manuscript. PG, EYW, RJ, NS, DZS, YS, and CNG reviewed and edited the manuscript. EYW acquired funding and was responsible for project administration.

## Funding support

This work is the result of NIH funding, in whole or in part, and is subject to the NIH Public Access Policy. Through acceptance of this federal funding, the NIH has been given a right to make the work publicly available in PubMed Central.

- NIH NHLBI and R01 HL152236 and R03HL146881.
- The Dean’s Research Fellowship of Columbia University (PG).
- The studies presented in this work were carried out in part in the MR Facility of the Oncology Precision Therapeutics and Imaging Core (OPTIC) Shared Resource, which is supported by funds from the Columbia University Medical Center Cancer Center Support Grant (CCSG) and NIH grant P30 CA013696 (National Cancer Institute).

## Supplementary Material

Supplemental data

Unedited blot and gel images

Supporting data values

## Version 1 10/07/2025

In-Press Preview

## Version 2 11/24/2025

Electronic publication
