# Supplementary material for: Atrial fibrillation–induced neurocognitive and vascular dysfunction is averted by mitochondrial oxidative stress reduction
Source: JCI Insight. 2025 Oct 7;10(22):e189850. doi: 10.1172/jci.insight.189850 (PMC12643510; doi:10.1172/jci.insight.189850)
Supplement: Supplemental data [file jciinsight-10-189850-s071.pdf]

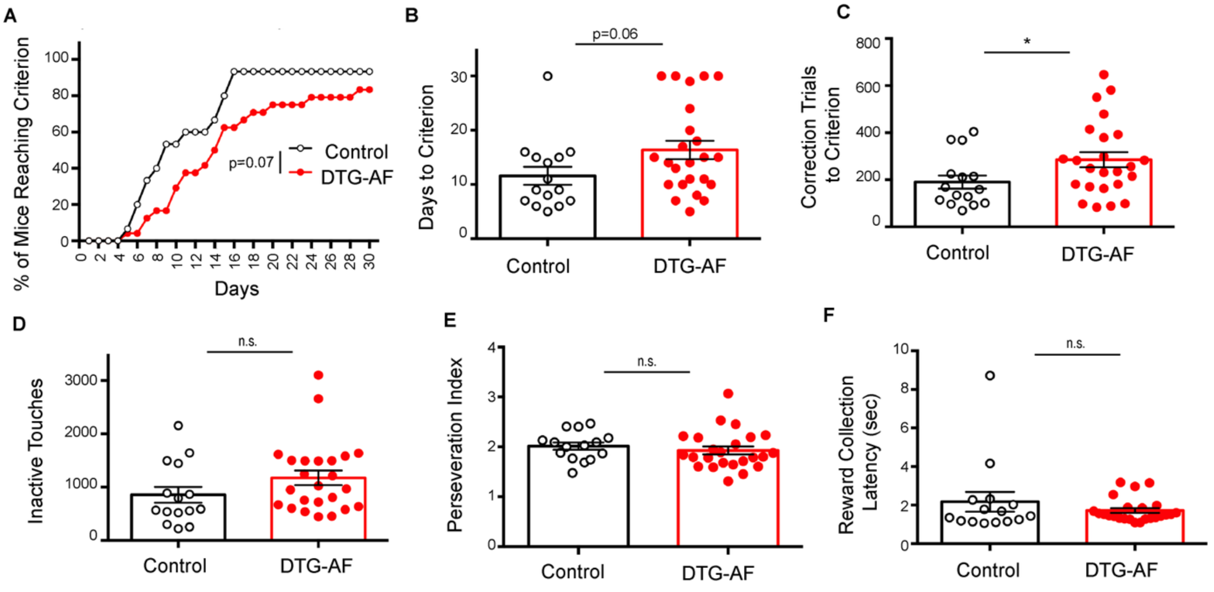

Supplemental Figure 1: Additional Acquisition Learning Results from Touchscreen Testing

(A) Percent of mice reaching criterion at each day, with DTG-AF showing a non-significant trend towards fewer mice reaching criterion across training than control (log-rank Mantel-Cox, $X^2 = 3.14$, $p = 0.077$) (n = 15 control, n = 24 DTG-AF mice). (B) Trend towards increased days to criterion in DTG-AF mice (unpaired t-test). (C) DTG-AF mice require more correction trials to reach criterion ($p < 0.05$). (D) DTG-AF mice show no significant differences compared with controls in (A) inactive touches during learning compared with controls (p = NS) (E) perseveration index representing number of continued incorrect responses after correction trial initiation (p = NS) and (F) latency to collect reward (p = NS). Data mean ± SEM. * $p < 0.05$.

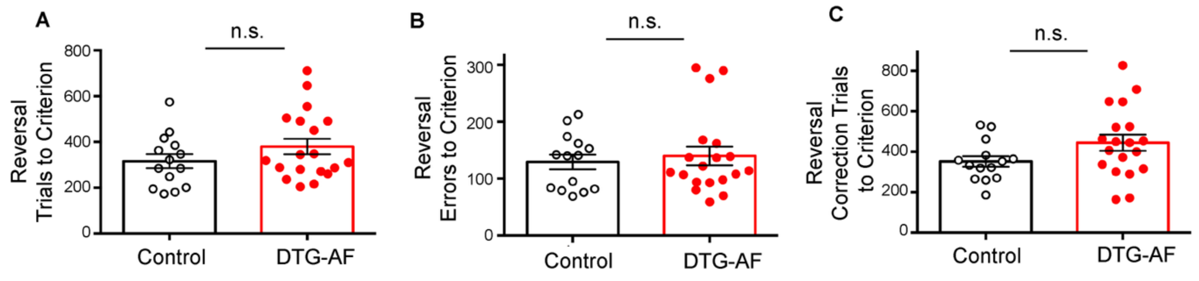

Supplemental Figure 2: Additional Reversal Learning Results

Reversal learning testing of DTG-AF mice does not show significant differences in (A) number of trials to achieve reversal criterion (p = NS) (B) number of errors made until criterion is reached (p = NS) or (C) total number of correction trials required during learning (p = NS). Data mean ± SEM.

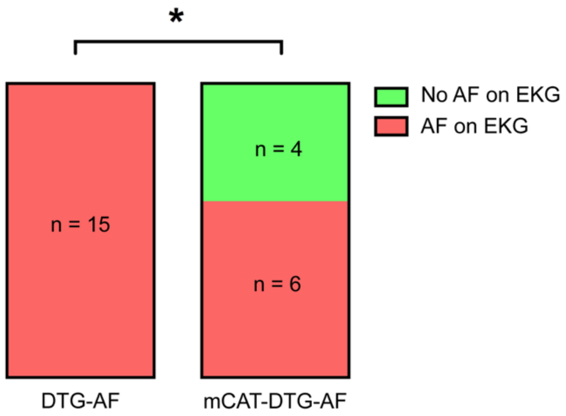

Supplemental Figure 3: Reduced AF in mCAT-DTG-AF mice

Fraction of mCAT-DTG-AF mice positive for AF on 2-minute ECG recordings is significantly lower than in DTG-AF mice (Fisher's exact test, * $p < 0.05$) (DTG-AF data same as Figure 1B).

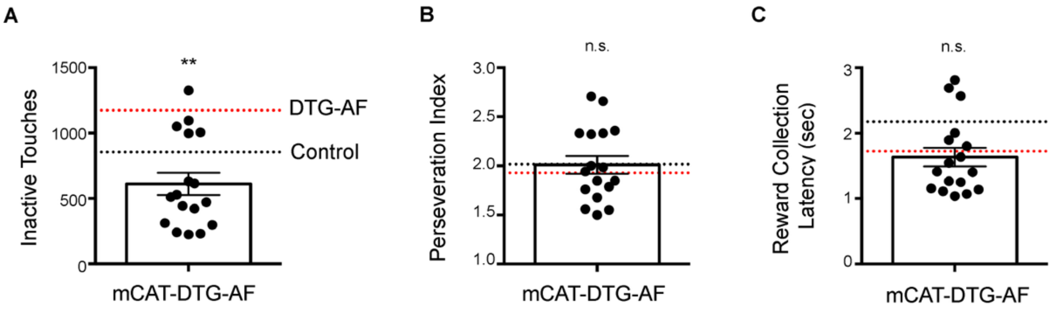

Supplemental Figure 4: Additional Acquisition Learning Results from Touchscreen Testing with mCAT-DTG-AF mice

Data from Supplemental Figure 1 visualized as dashed lines depicting the means of control and DTG-AF groups alongside mCAT-DTG-AF mice. Asterisks represent comparison between mCAT-DTG-AF and DTG-AF groups. (A) mCAT expression significantly improves the number of inactive touches made during learning compared to DTG-AF mice. One-way ANOVA and Tukey's test: DTG-AF vs mCAT-DTG-AF, $p < 0.01$; mCAT-DTG-AF vs. control, p = NS. (B) Perseveration index is no different between groups. (C) Reward collection latency is not significantly different between control, DTG-AF, and mCAT-DTG-AF mice. Data mean ± SEM, ** $p < 0.01$.

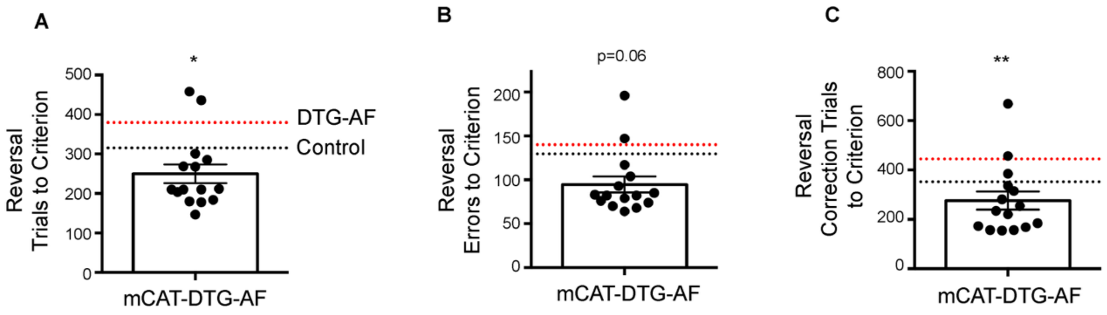

Supplemental Figure 5: Additional Reversal Learning Results with mCAT-DTG-AF mice

Data from Supplemental Figure 2 visualized as dashed lines depicting the means of control and DTG-AF groups alongside mCAT-DTG-AF mice. Asterisks represent comparison between mCAT-DTG-AF and DTG-AF groups. (A) mCAT expression significantly improves number of trials required to learn reversal criterion compared to DTG-AF mice. Tukey's test: DTG-AF vs. mCAT-DTG-AF, $p < 0.05$; mCAT-DTG-AF vs. control, p = NS. (B) mCAT expression trends towards reducing number of errors made during reversal learning compared to DTG-AF mice. Tukey's test: DTG-AF vs. mCAT-DTG-AF, $p = 0.06$; mCAT-DTG-AF vs. control, p = NS. (C) Reduced number of correction trials required in mCAT-DTG-AF mice compared to DTG-AF mice. Tukey's test: DTG-AF vs. mCAT-DTG-AF, $p < 0.01$; mCAT-DTG-AF vs control p = NS. Data mean ± SEM, * $p < 0.05$, ** $p < 0.01$.

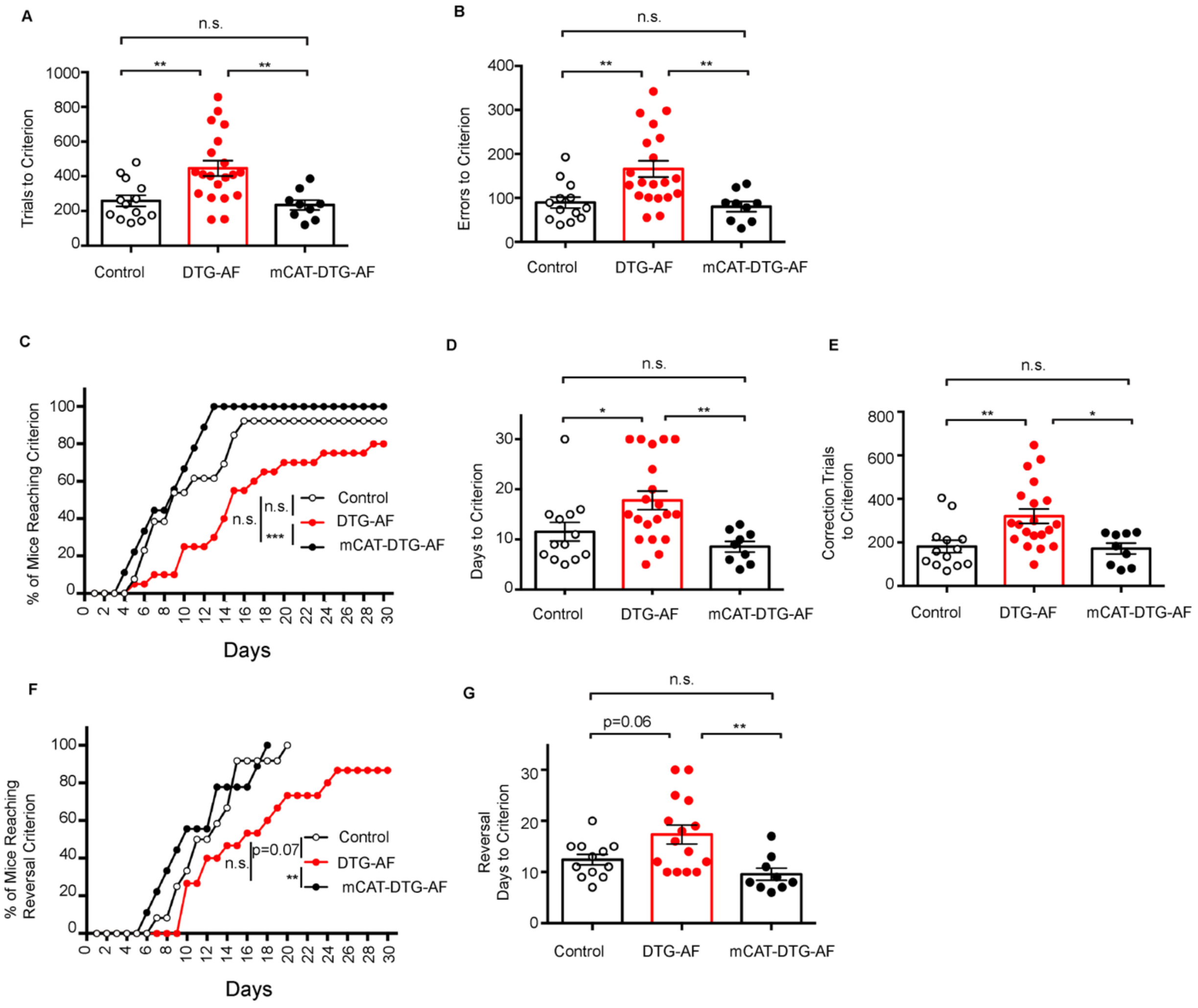

Supplemental Figure 6: Touchscreen Cognitive Testing in Mice 2-4 Months Old

Analysis of mice in the age group of 2-4 months demonstrates the same pattern of worse cognitive performance in DTG-AF mice than control, which is improved with expression of mCAT. (A-B) DTG-AF mice 2-4 months old require significantly greater trials and make more errors before reaching criterion performance, which is corrected in mCAT-DTG-AF mice (one-way ANOVA with Tukey's test, control vs DTG-AF $p < 0.01$, DTG-AF vs mCAT-DTG-AF $p < 0.01$, control vs mCAT-DTG-AF p = NS) (n = 13 control, n = 20 DTG-AF and n = 9 mCAT-DTG-AF mice). (C) Percent of mice reaching criterion at each day, with DTG-AF trending towards fewer mice reaching criterion across training than control (log-rank Mantel-Cox, $X^2 = 4.50$, $p = 0.03$) and mCAT-DTG-AF mice showing improved performance than DTG-AF (log-rank Mantel-Cox, $X^2 = 16.5$, $p < 0.0001$) (* $p < 0.02$, ** $p < 0.003$, *** $p < 0.0003$ per alpha corrected for multiple comparisons with Bonferroni's test). (D-E) Similar pattern of worse performance in DTG-AF improved by mCAT expression for days required to reach criterion and correction trials needed for learning. (F) Upon reversal of correct criterion, DTG-AF 2-4 month-old mice show trend of impaired reversal learning based on percent of mice reaching

criterion at each day (control vs DTG-AF log-rank Mantel-Cox, $X^2 = 4.98$, $p = 0.0257$) (DTG-AF vs mCAT-DTG-AF $X^2 = 11.7$, $p = 0.0006$) (** $p < 0.003$ per alpha corrected for multiple comparisons with Bonferroni's test) (n = 12 control, n = 15 DTG-AF and n = 9 mCAT-DTG-AF mice). (G) DTG-AF trend towards needing more days to learn the reversed criterion, which is corrected with mCAT expression. Data mean ± SEM, * $p < 0.05$, ** $p < 0.01$ except as otherwise noted.

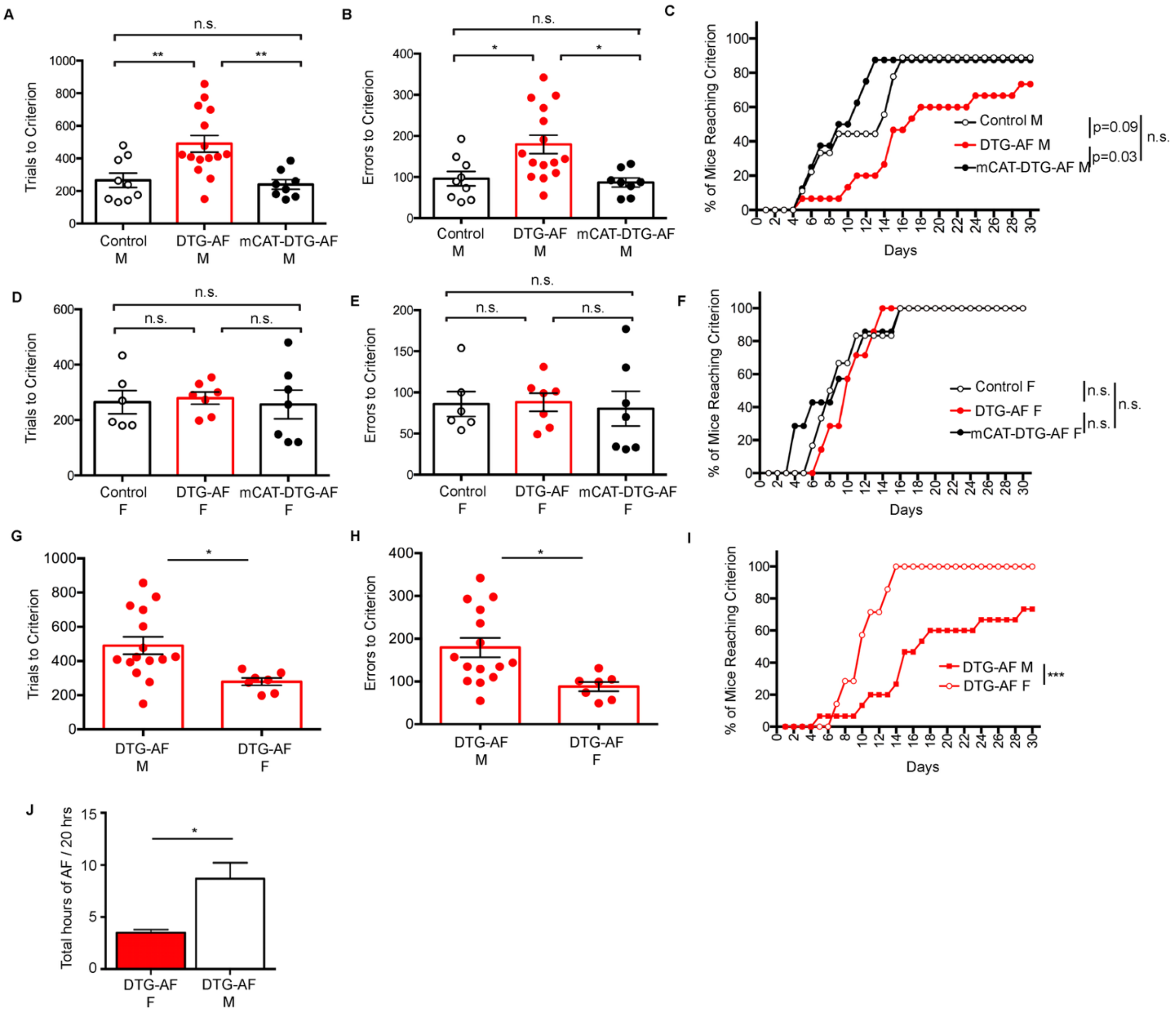

Supplemental Figure 7: Touchscreen Cognitive Testing in Male vs Female Mice

(A) Male DTG-AF mice require significantly greater trials to reach criterion than male control mice, which is corrected in male mCAT-DTG-AF mice (One-way ANOVA with Tukey's test, control vs DTG-AF $p < 0.01$, DTG-AF vs mCAT-DTG-AF $p < 0.01$, control vs mCAT-DTG-AF p = NS) (n = 9 control, n = 15 DTG-AF, n = 8 mCAT-DTG-AF male mice). (B) Male DTG-AF mice make significantly greater errors than control mice which is reversed with mCAT expression. (C) The percent of mice reaching criterion at each day shows a non-significant trend towards male DTG-AF mice requiring longer (log-rank Mantel-Cox, $X^2 = 2.88$, p = 0.089) and mCAT expression trending towards improving performance ($X^2 = 4.75$, p = 0.029) (* $p < 0.02$ per alpha corrected for multiple comparisons with Bonferroni's test). (D-F) Female DTG-AF mice do not show a significant difference in trials required to reach criterion or total errors (Tukey's test), or percent of mice reaching criterion each day (log-rank Mantel-Cox) (n = 6 control, n = 7 DTG-AF, and n = 7 mCAT-DTG-AF female mice). (G-H) Male DTG-AF mice require significantly greater trials to reach performance criterion than female DTG-AF mice and

make significantly more errors (unpaired t-test, $p < 0.05$). (I) Percent of male DTG-AF mice reaching criterion each day is significantly lower than female DTG-AF mice (log-rank Mantel-Cox, $X^2 = 12.4$, $p = 0.0004$). (J) Male DTG-AF mice show significantly greater burden of AF over a 20 hours period on implanted telemeters than age matched female DTG-AF mice (2.8 ± 0.5 vs. 8.5 ± 1.6 hrs, $p < 0.05$; n = 3 females, 5 males). Data mean ± SEM, * $p < 0.05$, ** $p < 0.01$, *** $p < 0.001$ except as otherwise noted.

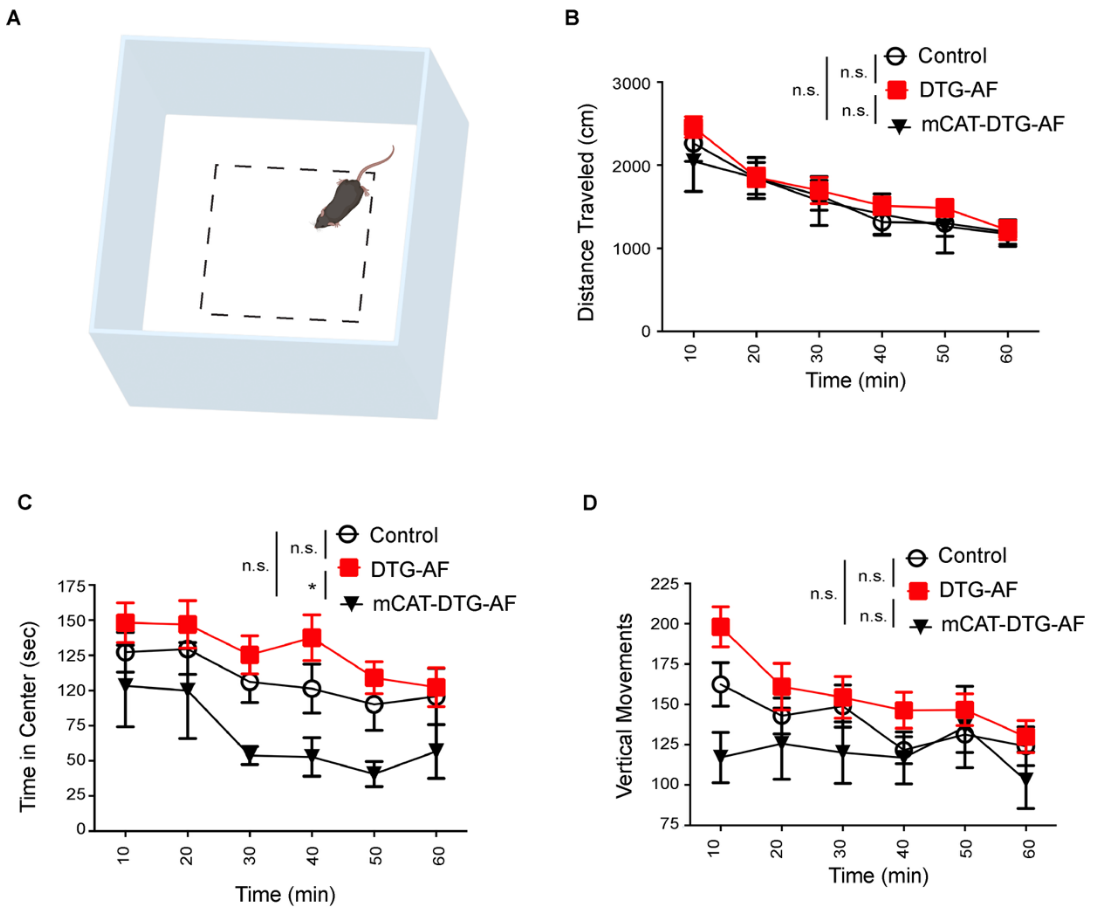

Supplemental Figure 8: Open Field Behavioral Testing in DTG-AF and mCAT-DTG-AF Mice

(A) Schematic depiction of mouse in open field arena. Dashed line demarcates exploratory center zone. (B) Total ambulatory distance traveled across time is no different between groups suggesting similar locomotor activity. Two-way repeated measures ANOVA, Tukey's test p = NS for all comparisons (n = 17 control, n = 13 DTG-AF, n = 6 mCAT-DTG-AF mice). (C) mCAT-DTG-AF mice spent significantly less time in the center of the arena than DTG-AF mice. Tukey's test DTG-AF vs. mCAT-DTG-AF, $p < 0.05$. (D) No significant differences in number of exploratory vertical movements between groups. Data mean ± SEM, * $p < 0.05$.

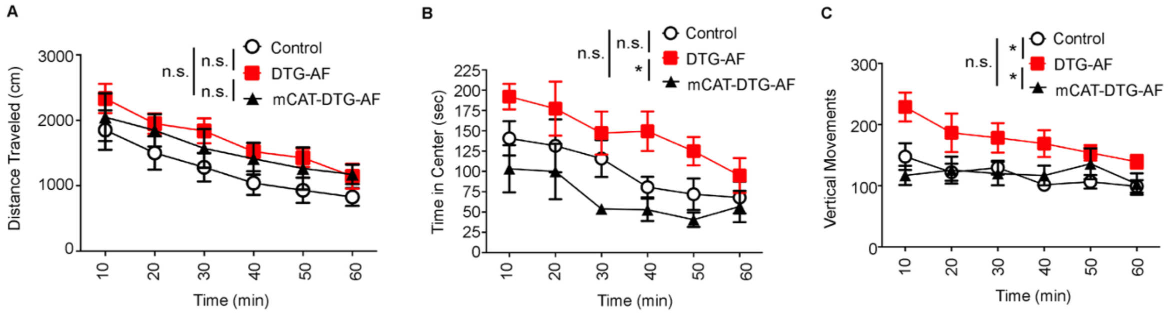

Supplemental Figure 9: Open Field Behavioral Results in Mice Older than 6 Months

(A) Distance traveled across time is not significantly different between groups, allowing for analysis without confounding by locomotor activity. Two-way repeated measured ANOVA, Tukey's test $p > 0.05$ for all comparisons. ($n = 9$ control, $n = 5$ DTG-AF, $n = 6$ mCAT-DTG-AF mice). (B) Aged DTG-AF mice trend towards greater time in center than control, and mCAT co-expression significantly reduces center time compared to DTG-AF mice. Tukey's test: control vs. DTG-AF, p = NS; DTG-AF vs. mCAT-DTG-AF, $p < 0.05$, mCAT-DTGAF vs. control, p = NS. (C) Aged DTG-AF mice show significantly greater vertical exploratory movements than control mice, which is corrected by mCAT co-expression. Tukey's test: control vs. DTG-AF, $p < 0.05$, DTG-AF vs. mCAT-DTG-AF, $p < 0.05$, mCAT-DTG-AF vs. control p = NS. Data mean ± SEM, * $p < 0.05$.

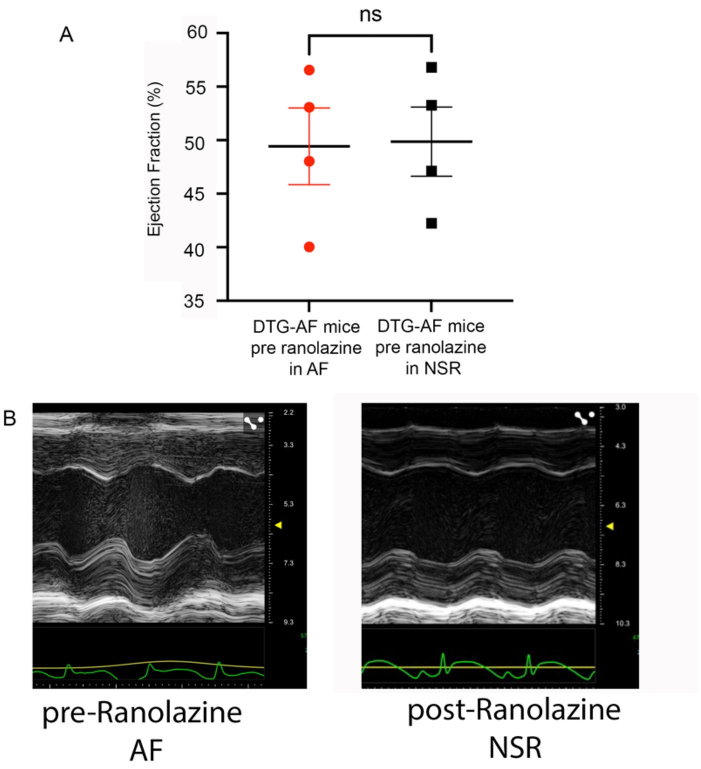

Supplemental Figure 10. Echocardiography to assess DTG-AF heart function in atrial fibrillation and post cardioversion to normal sinus rhythm. To assess whether the heart function of DTG-AF mice was affected by the heart rhythm: atrial fibrillation (AF) or normal sinus rhythm (NSR). We measured the left ventricular end diastolic diameter and left ventricular end systolic diameter using echocardiography. DTG-AF mice in AF before injection of ranolazine had an average LVEF of 49.42% vs an average LVEF 49.86% when in NSR post ranolazine, n=4 male DTG-AF mice 3 months of age. Data mean ± SEM, p = 0.93
