# Supplementary figures and images for: Atrial fibrillation–induced neurocognitive and vascular dysfunction is averted by mitochondrial oxidative stress reduction
Source: JCI Insight. 2025 Oct 7;10(22):e189850. doi: 10.1172/jci.insight.189850 (PMC12643510; doi:10.1172/jci.insight.189850)

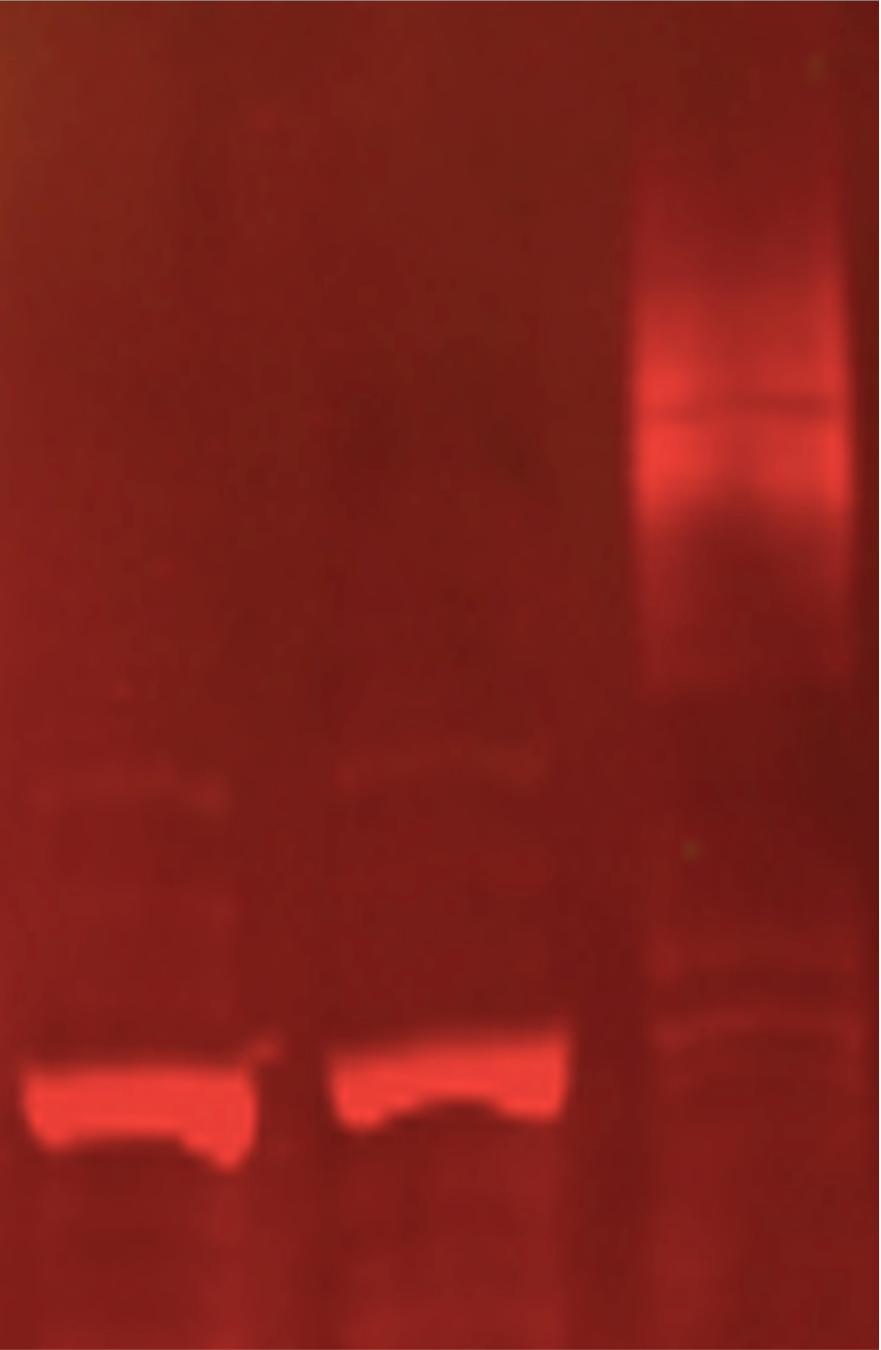

Supplement: Unedited blot and gel images [file jciinsight-10-189850-s072.pdf]
